# Gene and cell therapy of human genetic diseases: Recent advances and future directions

**DOI:** 10.1111/jcmm.70056

**Published:** 2024-09-08

**Authors:** Busra Cetin, Fulya Erendor, Yunus E. Eksi, Ahter D. Sanlioglu, Salih Sanlioglu

**Affiliations:** ^1^ Department of Gene and Cell Therapy, Faculty of Medicine Akdeniz University Antalya Turkey

**Keywords:** cell therapy, CRISPR/Cas9, gene editing, gene therapy, viral vectors

## Abstract

Disruptions in normal development and the emergence of health conditions often result from the malfunction of vital genes in the human body. Decades of scientific research have focused on techniques to modify or substitute defective genes with healthy alternatives, marking a new era in disease treatment, prevention and cure. Recent strides in science and technology have reshaped our understanding of disorders, medication development and treatment recommendations, with human gene and cell therapy at the forefront of this transformative shift. Its primary objective is the modification of genes or adjustment of cell behaviour for therapeutic purposes. In this review, we focus on the latest advances in gene and cell therapy for treating human genetic diseases, with a particular emphasis on FDA and EMA‐approved therapies and the evolving landscape of genome editing. We examine the current state of innovative gene editing technologies, particularly the CRISPR‐Cas systems. As we explore the progress, ethical considerations and prospects of these innovations, we gain insight into their potential to revolutionize the treatment of genetic diseases, along with a discussion of the challenges associated with their regulatory pathways. This review traces the origins and evolution of these therapies, from conceptual ideas to practical clinical applications, marking a significant milestone in the field of medical science.

## 1 INTRODUCTION TO GENE AND CELL THERAPY

The malfunction of vital genes in the body can lead to disruptions in normal development and the emergence of various health conditions. For decades, scientists have been researching techniques that involve modifying or substituting defective genes with healthy alternatives to treat, cure or prevent various diseases and medical conditions. Recent progress in science and technology has caused a shift in how we define disorders, develop medications and recommend treatments.

**FIGURE 1:**
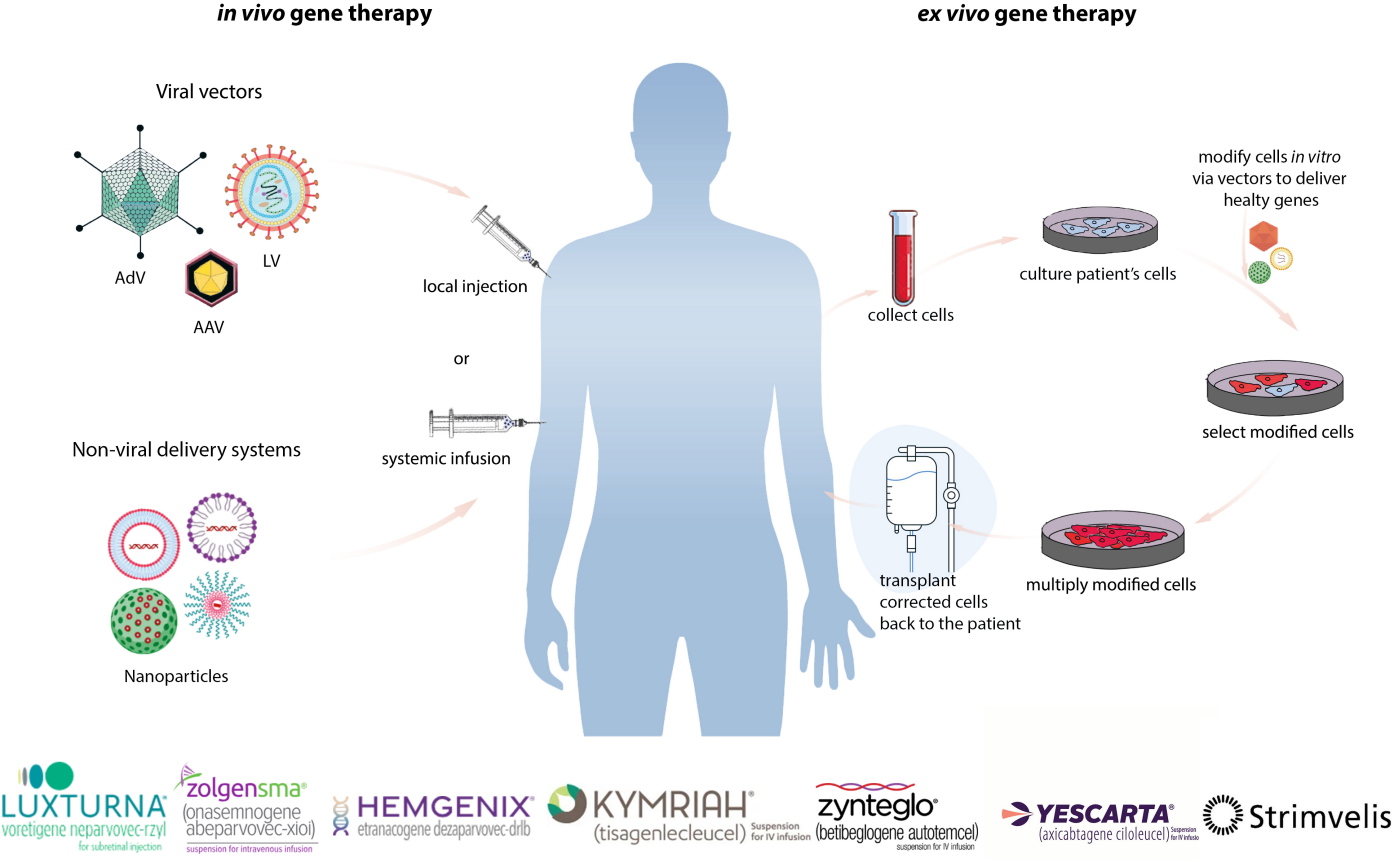
Ex vivo and in vivo somatic gene and cell therapy strategies. This figure illustrates two primary approaches to gene therapy: In vivo and ex vivo. In the in vivo gene therapy (left panel) strategy, a therapeutic gene is first inserted into a suitable vector either viral or non‐viral,^1^ most commonly employing Adeno‐associated viral (AAV) vectors. The vector, loaded with the therapeutic gene, is then introduced into the patient's body either via local injection or systemic infusion. The vector serves as a vehicle to deliver the therapeutic gene to the target cells within the patient's body. Ex vivo gene therapy (right panel) commences with the extraction of stem cells or other target cells from the patient's body. These isolated cells are then subjected to gene modification, often utilizing LVs. The therapeutic gene is introduced into the isolated cells outside the patient's body. After successful gene transfer, these modified cells are expanded and subsequently reintroduced into the patient. Representatives of commercially available gene therapy drugs are shown at the bottom.

Gene and cell therapies lie at the heart of this transformative shift, focusing on modifying genes or adjusting cell behaviour for therapeutic purposes. They are broadly categorized into two principal classes: Germline gene therapy (GGT), which involves modifications to the reproductive cell line and somatic cell gene therapy (SCGT), which focuses on the correction of genetic anomalies in non‐reproductive cells. While GGT holds significant promise, it remains ethically prohibited at present, precluding its practical application. Thus far, human gene therapy studies have primarily concentrated on SCGT, a field that has witnessed remarkable advancements. In the context of these therapies, cells can undergo modification either ex vivo before subsequent administration to humans or in vivo through direct gene therapy administration (Figure 1).^2^ In in vivo gene therapy, the viral/non‐viral vector carrying the therapeutic gene is introduced into the body via local or systemic injections. Ex vivo gene therapy starts with the extraction of the cells to be manipulated, such as stem cells/T‐cells, from the patient's body. The therapeutic gene is then transferred to the patient's cells outside the body via an appropriate vector, and the modified cells are reintroduced into the patients.

**FIGURE 2:**
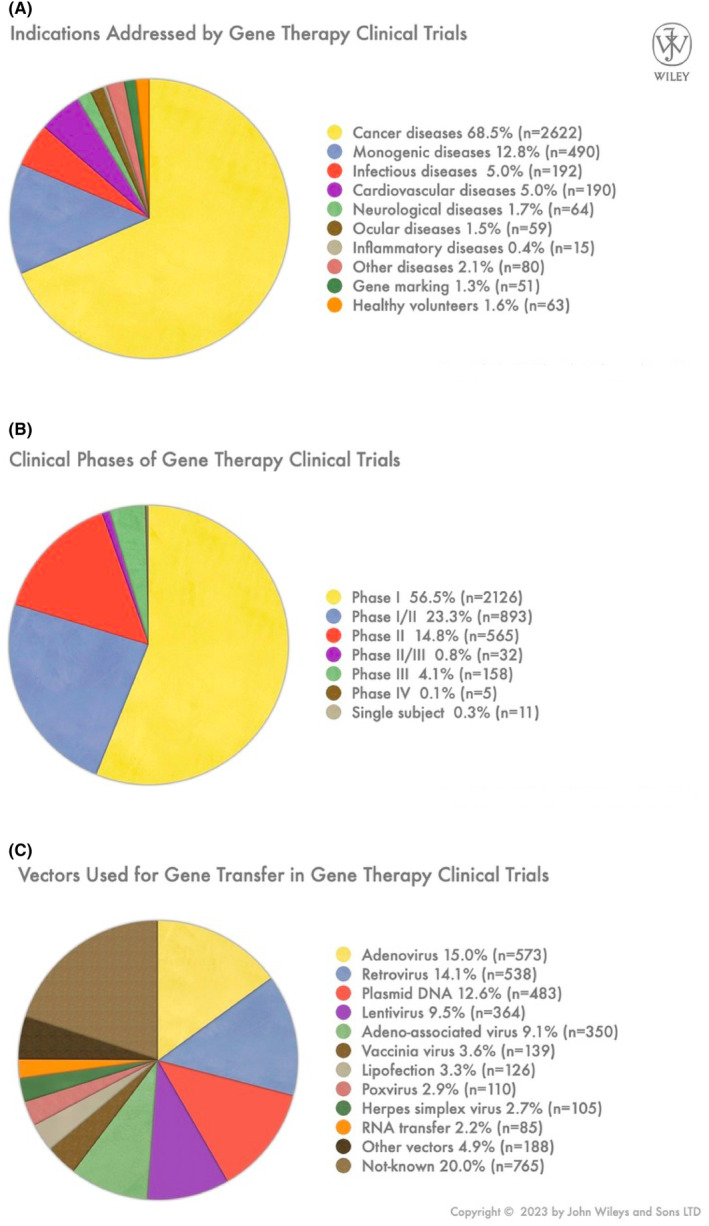
Current status of gene and cell therapy (As provided by The Journal of Gene Medicine Clinical Trial Website). Medical indications targeted by gene therapy clinical trials (A). This chart showcases the distribution of medical indications that gene therapy clinical trials aim to address. Various conditions, such as monogenic disorders, cancer, cardiovascular diseases, neurodegenerative disorders and others, are being targeted for potential therapeutic interventions through gene therapy research and trials. Clinical phases in gene therapy clinical trials (B). This chart displays the segmentation of gene therapy clinical trials based on their respective clinical phases. The phases include Phase I, Phase I‐II, Phase II, Phase II‐III, Phase III, Phase IV, and single subject. Each phase represents a distinct stage of testing, ranging from early safety assessments to large‐scale efficacy studies and regulatory approval. Vectors employed in gene transfer for gene therapy clinical trials (C). This chart illustrates the distribution of vector types utilized for gene transfer in various gene therapy clinical trials. Different vector platforms, such as viral vectors (adenovirus, adeno‐associated virus, lentivirus, retrovirus) and non‐viral vectors (nanoparticles), have been employed to deliver therapeutic genes.

Cellular and gene therapies have evolved to encompass a wide range of conditions, including single‐gene disorders, polygenic disorders, various forms of cancer, vascular diseases, neurodegenerative disorders, inflammatory conditions and acquired diseases. Numerous ongoing clinical studies continue to expand the spectrum of target diseases.^3^ As of 2023, gene therapy clinical trials have primarily focused on cancer, which constitutes the target of 68.5% of the studies (Figure 2A). Inherited monogenic diseases account for 12.8% and have seen notable success. Infectious diseases (5%) and cardiovascular diseases (5%) have also garnered attention. Beyond these, gene therapy is explored for various other conditions including rare diseases, highlighting its versatility.

Gene therapy clinical trials are predominantly in their initial phases (Figure 2B). Phase I trials account for the majority at 56.5%, closely followed by Phase I/II trials at 23.3%. Phase II trials make up 14.8% of all trials, with Phase II/III and Phase III combined representing a smaller portion at just 5%. However, there is a noteworthy trend towards new therapeutic areas, indicating progress in the field. In 2023, trials progressing to Phase II, II/III and III have reached 21.9%, suggesting ongoing advancements in gene therapy research that are bringing us closer to routine clinical integration. This early‐phase emphasis reflects the cautious and methodical approach taken in developing gene therapies, which ultimately plays a crucial role in optimizing the effectiveness of the specialized vehicles engineered to deliver the requisite genes for targeted disease treatment.^4^

As essential tools of gene therapy, vectors transfer genetic material (DNA or RNA) to target cells, with instructions to modulate the cellular production of a protein or a group of proteins. Vectors for gene delivery are traditionally categorized as viral and non‐viral vectors.^3^ Gene therapy trials have featured a wide spectrum of vectors and delivery techniques, with viral vectors being employed in approximately two‐thirds of trials as of 2023 as the predominant choice (Figure 2C). Adenoviral vectors (AdVs) lead the way at 15%, followed by retroviral vectors (RVs) at 14.1%. Notably, plasmid DNA has gained traction, with a usage rate of 12.6%. Additionally, lentiviral vectors (LVs) and adeno‐associated viruses (AAVs) are notable contenders at 9.5% and 9.1%, respectively. Clinical studies with other viral vectors, including vaccinia virus, herpes simplex virus and poxvirus, collectively account for 10.2% of all trials. Non‐viral approaches, such as lipofection and RNA transfer, also make their mark in the gene therapy landscape, collectively representing 5.5% of trials. These evolving trends reflect the dynamic nature of vector choices, continually shaping the field of gene therapy research.

**FIGURE 3:**
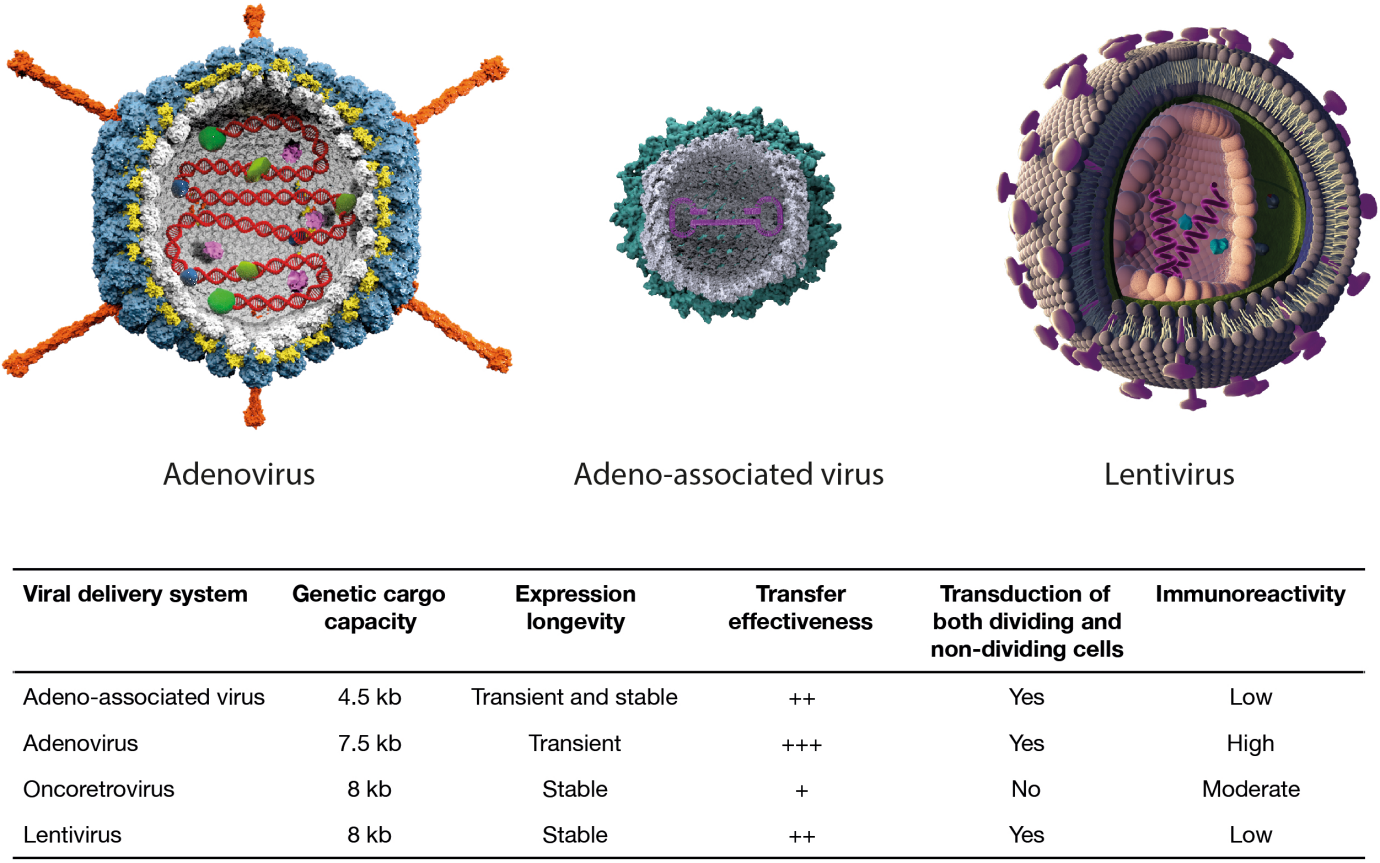
Types of viral vector platforms and their inherent traits employed for genetic transfer.^5^, ^6^ The table lists diverse vector types (adenovirus, adeno‐associated virus, lentivirus, and retrovirus) utilized in clinical and preclinical investigations in terms of expression longevity, transduction ability, packaging capacity, and immunogenic response.

Viral vectors have garnered prominence as efficient carriers in Food and Drug Administration (FDA)‐approved cellular and gene therapies (Figure 3). LVs, originating from the human immunodeficiency virus (HIV), play a crucial role in the field of gene and cell therapy predominantly due to their safe nature, and ability to infect dividing and non‐dividing cells to integrate therapeutic genetic material into the host cell's genome. This integration ensures the stable, long‐term expression of the desired gene, making them particularly well‐suited for addressing diseases such as diabetes, Parkinson's disease and genetic conditions like haemophilia that require continuous production of specific proteins.^7^, ^8^ On the other hand, AdVs, derived from adenoviruses, are highly regarded for their exceptional transduction efficiency into a variety of cell types, rendering them invaluable tools in the field of gene therapy.^9^, ^10^ In contrast, AAV has been a promising gene therapy vector, despite its limitations in its target tissue range, primarily muscle and the brain. Recent molecular studies have expanded our understanding of the AAV transduction mechanisms, enabling the development of innovative strategies to overcome intracellular barriers. AAV vectors stand out by having the highest number of approved gene therapy drugs. This achievement underscores their demonstrated effectiveness and safety in clinical practice.^11^, ^12^

In parallel, non‐viral methodologies (plasmid DNA, bacterial vectors, human gene editing tools and patient‐derived cellular gene therapy products) are undergoing extensive investigation as a promising avenue for safe and efficacious delivery of genetic material to target cells. In spite of significant efforts in developing viral and non‐viral gene delivery systems over the past three decades, these systems still exhibit limitations that hinder their broad clinical application. Currently, there is no universal delivery system capable of effectively targeting all cell types in both ex vivo and in vivo settings without limitations or side effects. While certain delivery systems show promise for specific cell or tissue targeting, the development of successful delivery systems, particularly non‐viral ones for in vivo applications, is still in its early stages.

In this review, we discuss the landscape of approved gene and cell therapy products, exploring their target indications, primary clinical trial outcomes, safety profiles, manufacturing processes, recommended dosages and sales figures (Table 1). These products represent a significant advancement in the field of medicine, offering innovative solutions for various medical conditions. Understanding their characteristics and impact is essential for evaluating their potential benefits and limitations in clinical practice.

**TABLE 1:** Overview of approved gene and cell‐based products as of 2023.

Trade name	Indications	Approving agency, approval year	Properties	Manufacturer
Gendicine®	Head and neck squamous cell carcinoma	CFDA, 2003	AdV	Shenzhen SiBiono GeneTech Co. Ltd
Oncorine® (H101)	NPC and head and neck cancer	CFDA, 2005	AdV	Shanghai Sunway Biotech
Glybera® (alipogene tiparvovec)	Familial lipoprotein lipase deficiency	EMA, 2012	AAV	UniQure biopharma B.V.
IMLYGIC® (talimogene laherparepvec)	Melanoma lesions	FDA, 2015; EMA, 2015	HSV	BioVex Inc., a wholly owned subsidiary of Amgen, Inc.
Strimvelis®	ADA‐SCID	EMA, 2016	RV; ex vivo	Orchard Therapeutics B.V.
Kymriah® (tisagenlecleucel)	Acute lymphoblastic leukaemia	FDA, 2017; EMA, 2018	LV; ex vivo	Novartis Pharmaceuticals Corporation
Luxturna® (voretigene neparvovec‐rzyl)	Leber congenital amaurosis	FDA, 2017; EMA, 2018	AAV	Spark Therapeutics, Inc.
Zolgensma® (onasemnogene abeparvovec)	Spinal muscular atrophy	FDA, 2019; EMA, 2022	AAV	Novartis Gene Therapies, Inc.
Zynteglo® (betibeglogene autotemcel)	Beta‐thalassamia	EMA, 2019; FDA, 2022	LV; ex vivo	Bluebird Bio, Inc.
Hemgenix® (etranacogene dezaparvovec)	Haemophilia B	FDA, 2022; EMA, 2022	AAV	CSL Behring LLC
Adstiladrin® (nadofaragene firadenovec)	High‐risk BCG‐unresponsive NMIBC	FDA, 2022	AdV	Ferring Pharmaceuticals A/S
Casgevy® (exagamglogene autotemcel)	Sickle cell disease (SCD)	FDA, 2023	CRISPR	Vertex Pharmaceuticals Incorporated
Lyfgenia® (lovotibeglogene autotemcel)	Sickle cell disease (SCD)	FDA, 2023	LV	Bluebird bio, Inc.

Abbreviations: AAV, adeno‐associated virus; AdV, adenovirus; CFDA, China Food and Drug Administration; EMA, European Medicines Agency; FDA, US Food and Drug Administration; HSV, herpes simplex virus; LV, lentivirus; RV, retrovirus.

## 2 GENDICINE®: FIRST COMMERCIALLY APPROVED GENE THERAPY PRODUCT

In the field of gene therapy, pioneering products have paved the way for personalized and targeted treatments for cancer. Gendicine® is a genetically modified E1‐deleted adenovirus designed to express wildtype p53. It is the world's first gene therapy product developed by Shenzhen SiBiono GeneTech, which received regulatory approval from the Chinese State Food and Drug Administration (CFDA) in 2003. Gendicine's primary focus was addressing the challenges of head and neck squamous cell carcinoma (HNSCC), particularly in patients with mutated p53 genes. Gendicine's clinical trajectory spans over 12 years, involving more than 30,000 patients and a wealth of published clinical studies.^13^ Its safety record is exemplary, especially when utilized alongside chemotherapy and radiotherapy, consistently delivering response rates that surpass standard therapies. Beyond HNSCC, Gendicine has showcased its potential in treating a spectrum of advanced cancers, including lung cancer, liver cancer, gynaecological tumours and soft tissue sarcomas. A testament to its impact, 13 published studies have provided long‐term survival data, with combination regimens demonstrating significantly prolonged progression‐free survival times compared to standard therapies.^14^

## 3 ONCORINE®: ADVANCING ONCOLYTIC GENE THERAPY THROUGH ADENOVIRAL PRECISION

Oncorine®, also known as H101, is a type of oncolytic virus therapy used in the treatment of cancer. It is a new class of therapeutic agents that are designed to be capable of both direct tumour cell eradication and activation of host anti‐tumour immune responses. Within this category, various viruses such as adenovirus, autonomous parvoviruses, vaccinia virus and vesicular stomatitis virus have been harnessed. These oncolytic viruses exhibit the ability to selectively replicate within cancer cells, culminating in tumour lysis. Simultaneously, the release of infectious viral particles (vp) from lysed tumour cells perpetuates the attack on remaining cancerous tissue. The release of tumour‐associated antigens into the local microenvironment incites a robust immune reaction.^15^, ^16^

Oncorine® achieved the distinction of being the inaugural oncolytic virus to receive approval from the CFDA in November 2005. Developed by Shanghai Sunway Biotech in China, Oncorine® is a genetically modified adenovirus serotype 5 named H101 (E1B‐deletion). Its innovative application entails pairing with chemotherapy for the treatment of nasopharyngeal carcinoma (NPC) and head and neck cancer. This approval and successful utilization of Oncorine® have boosted the advancement of cancer immunotherapy grounded in oncolytic virus strategies. Oncorine® has shown safety and efficacy in clinical investigations, offering enhanced tumour‐killing effects without the high toxicity of chemotherapy. Despite this groundbreaking achievement, Oncorine's market reach remains confined within China, and the USA's analogous Onyx‐15 virus encountered an early‐phase III trial suspension due to funding constraints.^17^

## 4 GLYBERA®: FIRST EMA‐APPROVED GENE THERAPY DRUG

Glybera®, known by several names such as AMT‐011, and AAV1‐LPL S447X, represents a pioneering advancement in gene therapy. This innovative medication is administered via intramuscular injections and harnesses the power of a modified AAV1 virus to deliver the lipoprotein lipase gene into cells. Specifically designed for adults dealing with lipoprotein lipase deficiency, even in the face of stringent dietary measures, Glybera's injections trigger muscle cells to produce the crucial missing enzyme. This enzymatic boost significantly reduces the severity of pancreatitis, a painful inflammation of the pancreas, which can be debilitating for those afflicted. An essential aspect of Glybera's treatment protocol involves immunosuppression, both before and following administration, to mitigate any adverse immune responses. This advanced therapy's modified viral material is non‐infectious, ensuring the safety of the treatment process.

Furthermore, Glybera's exclusivity is underscored by its strict patient criteria. It is solely intended for individuals with confirmed lipoprotein lipase deficiency, a diagnosis established through genetic testing and the presence of detectable lipoprotein lipase enzyme levels in their blood. Glybera® was classified as an ‘orphan medicine’ in 2004, in recognition of the rarity of the condition it addresses, and the limited number of people affected by it. This classification acknowledges the unique challenges faced by those with rare diseases and underlines the need for specialized treatments. The therapy is available only through a prescription and must be administered under the expert guidance of a specialist well‐versed in lipoprotein lipase deficiency and gene therapy. Remarkably, Glybera® offers a one‐time treatment solution designed to provide relief for at least a decade.^18^

Glybera's approval is a significant milestone in the realm of gene therapy. It received a favourable recommendation from EMA in July 2012, followed by approval from the European Commission in November of the same year. This achievement marked a historic moment as Glybera became the first gene therapy treatment to be authorized in Europe, setting a precedent for future gene‐based therapies.^19^ Nonetheless, despite its groundbreaking status, Glybera® faced challenges related to its cost‐effectiveness. With a staggering price tag exceeding €1 million per patient, it emerged as one of the most expensive therapies ever approved in Europe. This cost, coupled with the rarity of lipoprotein lipase deficiency, limited its commercial success. Furthermore, Glybera's inability to secure approval in the United States led to uniQure's decision to withdraw the drug from the European market in 2017 due to unsustainable manufacturing expenses and financial losses. To date, only 31 individuals worldwide have benefited from Glybera®, and its availability remains absent in the United States and Canada.^18^, ^20^

## 5 IMLYGIC® (TALIMOGENE LAHERPAREPVEC; T‐VEC): MELANOMA TREATMENT AND THE PROMISE OF ONCOLYTIC VIROTHERAPY

Melanoma accounts for a small percentage of skin cancer cases but is responsible for a disproportionate number of skin cancer‐related deaths. Its high aggressiveness, resistance to chemotherapy, and potential for metastasis have made it one of the most challenging cancers to treat. Traditional treatment modalities, such as surgery, radiation and chemotherapy, have shown limited success in advanced cases.^21^ However, recent breakthroughs in immunotherapy, particularly oncolytic virotherapy, have provided a ray of hope for patients with melanoma.

Talimogene laherparepvec, commonly referred to as T‐VEC®, represents a novel class of immunotherapeutic agents designed to target melanoma lesions. It belongs to the category of oncolytic viruses, which are genetically modified viruses engineered to infect and replicate within cancer cells while sparing normal, healthy cells. T‐VEC® is derived from a modified strain of herpes simplex virus 1 (HSV‐1). Notably, it lacks a critical gene known as ICP34.5, rendering it replication‐competent only within tumour tissue.^22^ This unique feature allows T‐VEC® to selectively target melanoma lesions.

T‐VEC's mode of action involves a two‐pronged attack on melanoma. First, when injected directly into visible or ultrasound‐detected tumours, T‐VEC® enters cancer cells and starts replicating. As these infected cells multiply, they ultimately burst, leading to the release of numerous copies of the virus. This process effectively lyses the tumour cells, causing the tumour to break down. Second, T‐VEC® is engineered to express granulocyte‐macrophage colony‐stimulating factor (GM‐CSF), a substance that plays a key role in immune response activation. GM‐CSF enhances the body's natural defences against cancer cells by recruiting immune cells to the site of infection, where they can recognize and attack the tumour. This dual mechanism of tumour cell destruction and immune system activation makes T‐VEC® a potent weapon against melanoma.^22^, ^23^

The clinical efficacy of T‐VEC® was demonstrated in the OPTiM trial, a landmark study that evaluated its effectiveness in advanced melanoma patients. The trial revealed a notable 31.5% response rate in patients treated with T‐VEC®, with a significant survival benefit compared to conventional therapies. These results prompted FDA to grant approval for T‐VEC® in the treatment of advanced melanoma in 2015. This historic approval marked the first endorsement of an oncolytic virus therapy in Western medicine.^24^ Patients treated with T‐VEC® may experience manageable side effects such as fatigue, chills, fever, nausea and flu‐like symptoms, which are outweighed by the therapy's potential benefits. The success of T‐VEC® in melanoma has sparked interest in exploring its application in other cancers, as well as in combination therapies. Ongoing research seeks to uncover the full potential of oncolytic virotherapy in the broader landscape of cancer treatment.^25^, ^26^, ^27^

## 6 GENE THERAPY BREAKTHROUGH FOR ADA‐SCID: STRIMVELIS® AND BEYOND

Adenosine Deaminase Severe Combined Immunodeficiency Disorder (ADA‐SCID), colloquially referred to as bubble‐boy disease, disrupts the immune system profoundly, leaving individuals vulnerable to life‐threatening infections. This rare inherited disorder results from a mutation in the adenosine deaminase gene, pivotal for producing healthy lymphocytes. As ADA‐SCID patients lack functional immune responses, survival without intervention is limited to approximately 2 years. Rooted in an inherited gene defect, the condition hampers adenosine deaminase synthesis, leading to toxic deoxyadenosine accumulation that detrimentally affects infection‐fighting lymphocytes. Children afflicted by ADA‐SCID endure compromised immunity, growth impediments, hearing impairment and organ complications, often culminating in fatality within the initial years of life.

Restoring immune function in ADA deficiency entails bone marrow transplantation, enzyme replacement, and gene therapy. The effectiveness of these approaches varies based on treatment strategies and patient‐specific attributes. Transplants from genetically matched siblings offer improved outcomes, whereas transplants from unrelated donors carry the risk of graft versus host disease and heightened susceptibility to infections due to immunosuppression.^28^ While enzyme replacement therapy utilizing pegylated adenosine deaminase (PEG‐ADA) is sporadically employed, concerns linger about sustained immune decline. Innovative gene therapy involving autologous haematopoietic stem cells transduced with ADA cDNA presents a pioneering avenue, yielding diverse outcomes in immune reconstitution and potential post‐treatment autoimmune responses.^29^

Strimvelis®, a groundbreaking treatment developed by GlaxoSmithKline (GSK) at the San Raffaele Telethon Institute for Gene Therapy, revolutionizes therapy by incorporating a functional adenosine deaminase enzyme gene into immature bone marrow cells (CD34+ cells) from the patient.^30^ Following this genetic enhancement, the modified cells are reintroduced into the patient's body, where they differentiate into various blood and immune cell types. This innovative process bestows patients with a lifelong ability to generate infection‐fighting lymphocytes, circumventing graft versus host disease risks and minimizing infection susceptibility associated with immunosuppression. Furthermore, this treatment significantly reduces the required chemotherapy dosage compared to conventional bone marrow transplants. An essential advantage of gene therapy is the elimination of the challenges posed by donor searches, making this approach universally applicable.^31^

EMA's endorsement of Strimvelis®, the first approved ex vivo autologous gene therapy in Europe in 2016 for ADA‐SCID cases lacking matched haematopoietic stem cell donors was grounded in a successful trial demonstrating a remarkable 100% survival rate over 7 years.^32^ Approximately 80% of patients lack matched donors. European Commission approval in May 2016, with a cost exceeding enzyme replacement therapy, positions Strimvelis® as a transformative solution for ADA‐SCID, an exceptionally rare disorder. The promise of gene therapy is undeniable, although the prospect of RVs activating cancer genes underscores the need for cautious exploration. Notably, the link between Orchard Therapeutics' Strimvelis® and a patient's leukaemia underscores ongoing investigation, as only 16 patients have received this treatment since 2016. Orchard's pursuit of OTL‐101, a safer lentivirus‐based alternative, reinforces their commitment to addressing safety concerns across their gene therapy portfolio, which primarily employs designed LVs to prevent oncogenesis.^31^, ^33^

## 7 KYMRIAH®: THE FIRST FDA‐APPROVED CAR‐T CELL THERAPY

Acute lymphoblastic leukaemia (ALL), also known as acute lymphocytic leukaemia, is a type of cancer that starts in the bone marrow. Specifically, ALL affects the lymphoblasts, a type of immature white blood cell or lymphocyte. In ALL, these lymphoblasts do not mature properly and become abnormal, rapidly multiplying leukaemia cells. These abnormal cells then crowd out and interfere with the production of normal white blood cells, red blood cells and platelets in the bone marrow.^34^ Immunotherapy using chimeric antigen receptors (CARs) shows promise in treating ALL. This technique involves combining elements from a T‐cell receptor and an antibody that recognizes the CD19 protein found on B‐cells. CD19 is a marker that distinguishes cancerous B‐cells from healthy ones, making it an ideal target for identifying potentially cancerous B‐cell populations. T‐cells are genetically modified to recognize CD19, resulting in a chimeric T‐cell receptor (CAR‐T) that is integrated into the T‐cell membrane.^35^ Various methods can be used to introduce the CAR‐encoding transgene into immune T‐cells, with LVs being the most common method. Self‐inactivating (SIN) lentiviruses are particularly effective in integrating the desired gene into target cells. Alternative methods like electroporation and transfection exist, but their efficiency is limited by decreasing transgene expression over time.^36^

Kymriah® is an autologous T‐cell immunotherapy that has gained regulatory approval for treating paediatric and young adult patients (up to 25 years old) with B‐cell ALL.^37^ Kymriah's treatment involves personalized administration using the patient's T‐cells. These T‐cells are collected and genetically modified in a specialized facility to include CAR protein. CAR enables the T‐cells to selectively recognize and eliminate leukaemia cells marked by CD19.^2^, ^37^ Notably, the CAR in Kymriah® incorporates the 4‐1BB co‐stimulatory domain for enhanced responsiveness. After genetic modification, the engineered T‐cells are reintroduced into the patient's body through infusion, enabling them to actively target and eliminate the cancerous cells.^38^, ^39^ It is intended for cases where cancer has not responded well to initial treatment or has relapsed, affecting about 15%–20% of cases.^37^ Additionally, Kymriah® is used in large B‐cell lymphoma and follicular lymphoma (both non‐Hodgkin lymphomas) when previous treatments have failed.^40^ The concept of Kymriah® was developed at the University of Pennsylvania and later advanced, endorsed by the FDA, and commercialized by Novartis. Notably, in August 2017, Kymriah® became the first FDA‐approved CAR‐T cell therapy in the United States, incorporating gene therapy in its treatment protocol.^41^

Kymriah® has been proven safe and effective in a clinical trial involving 63 paediatric and young adult patients with relapsed or refractory B‐cell precursor ALL. Within 3 months of treatment, 83% of patients achieved remission and after 1 year, 79% of patients remained in survival follow‐up.^38^ This is a significant improvement as conventional chemotherapy typically only has a survival rate of 16%–30% for patients with resistance.^34^, ^35^, ^41^, ^42^ However, it's crucial to recognize the inherent risks associated with Kymriah® therapy. Patients must be warned of the possibility of severe adverse effects, with cytokine release syndrome (CRS) and neurological events being the most common, caused by CAR T‐cell activation and proliferation. These symptoms include fever, flu‐like symptoms and neurological complications, which can be life‐threatening if not recognized and treated promptly.^43^ Other severe outcomes include severe infections, hypotension, kidney injury, fever and reduced oxygen levels. These effects may occur within 1–22 days after treatment. Additionally, Kymriah's targeting of CD19 antigens may make non‐malignant B‐cells vulnerable to prolonged infections, particularly with diffuse large B‐cell lymphoma.^38^

FDA‐approved CAR T‐cell therapies focus on antigens CD19 and BCMA, found in B cells. Since 2017, the FDA has approved six CAR T‐cell therapies (Table 2) for hematologic malignancies including lymphomas, specific leukaemias, and recently, multiple myeloma. This culmination of years of research has positioned CAR T‐cell therapies as a standard‐of‐care option for aggressive lymphomas, with global availability.^44^

**TABLE 2:** Details of six approved CAR‐T cell products.^44^

FDA‐approved CAR‐T cell therapies	Target antigen	Targeted disease
Kymriah® (tisagenlecleucel) ‐ Approved by FDA in 2017	CD19	B‐cell precursor acute lymphoblastic leukaemia (ALL) B‐Cell Non‐Hodgkin Lymphoma (NHL)
Yescarta® (axicabtagene ciloleucel) ‐ Approved by FDA in 2017	CD19	B‐Cell Non‐Hodgkin Lymphoma (NHL) Follicular Lymphoma
Tecartus® (brexucabtagene autoleucal) ‐ Approved by FDA in 2020	CD19	Mantle cell lymphoma (MCL) B‐cell precursor acute lymphoblastic leukaemia (ALL)
Breyanzi® (lisocabtagene maraleucel) ‐ Approved by FDA in 2021	CD19	B‐Cell Non‐Hodgkin Lymphoma (NHL)
Abecma® (idecabtagene vicleucel) ‐ Approved by FDA in 2021	BCMA	Multiple myeloma
Carvykti® (ciltacabtagene autoleucel) ‐ Approved by FDA in 2022	BCMA	Multiple myeloma

*Note*: This table provides information on six approved Chimeric Antigen Receptor T‐cell (CAR‐T cell) products and presents key details about each approved product, including the product name, targeted disease, and target antigen.

## 8 LUXTURNA®: PIONEERING GENE THERAPY FOR RPE65‐ASSOCIATED RETINAL DYSTROPHY

Retinal dystrophies constitute a heterogeneous group of ocular disorders characterized by the progressive degeneration of various retinal components, often stemming from mutations in diverse genes. Among the spectrum of inherited retinal dystrophies, Leber congenital amaurosis (LCA) emerges as a particularly severe and early‐onset variant, leading to congenital blindness. The term ‘congenital’ signifies conditions present from birth, while ‘amaurosis’ implies vision loss unrelated to any lesion.^45^ Despite these overarching characteristics, LCA exhibits a diverse manifestation due to its association with multiple genes. This syndrome typically presents with nystagmus, impaired pupillary responses and profound vision impairment or blindness. Its prevalence is estimated to affect approximately 1 in 40,000 newborns.^46^

A primary causative factor underlying these conditions is genetic mutations affecting the retinal pigment epithelium‐specific protein 65 kDa (RPE65) gene. Positioned within the retinal pigment epithelial cells, RPE65 plays a pivotal role in the conversion of all‐trans‐retinol into 11‐cis‐retinol.^47^ This conversion is integral to the generation of the visual chromophore, 11‐cis‐retinal, during the retinoid cycle. These events are crucial for the translation of light photons into electrical signals within the retina. The presence of mutations in the RPE65 gene results in the reduced or absent activity of RPE65 all‐trans‐retinyl isomerase, which subsequently disrupts the visual cycle and triggers vision impairment. With time, the accumulation of detrimental precursor molecules contributes to the degeneration of retinal pigment epithelial cells, leading to a progressive loss of photoreceptor cells.^48^, ^49^, ^50^, ^51^ Individuals carrying biallelic RPE65 mutations, which are associated with retinal dystrophy, experience a gradual loss of vision, typically emerging during childhood or adolescence. This deterioration in visual acuity and visual field parameters ultimately advances to a state of complete blindness.

The success of gene therapy in addressing RPE65‐associated retinal dystrophy is exemplified by Luxturna® (Voretigene neparvovec). This groundbreaking gene therapy utilizes an AAV2 vector and is designed for individuals experiencing vision loss due to inherited retinal dystrophy stemming from confirmed biallelic RPE65 mutations. Collaboratively developed by Spark Therapeutics and Children's Hospital of Philadelphia, Luxturna® received FDA approval on 19 December 2017.^51^ Administration of Luxturna® mandates the presence of viable retinal cells and involves a subretinal injection.^52^ Notably, this therapy has not only revolutionized the quality of life for individuals who previously faced the prospect of blindness but has also propelled the exploration of gene therapy targeting various other genetic forms of inherited retinal disease.

Clinical trials have unequivocally demonstrated the short‐term efficacy of Luxturna®, showcasing vision improvement and disease progression.^53^ Although long‐term clinical evidence has yet to fully materialize, the treatment's biological plausibility strongly suggests the potential for sustained benefits. While Luxturna® does not serve as a complete cure for the condition, it significantly enhances vision in treated individuals. However, it is prudent to note that Luxturna's application is not recommended for patients under 12 months of age due to ongoing retinal growth that could influence its effectiveness.^54^

An additional aspect to underscore is the economic dimension of Luxturna® therapy, which comes with a substantial cost of approximately $850,000 per therapy (equivalent to $425,000 per eye for bilateral treatment), which has sparked discussions on accessibility and broader implications for gene therapies.^55^

## 9 ZOLGENSMA®: REVOLUTIONIZING SMA TREATMENT—FROM GENETIC BASIS TO ADVANCED GENE THERAPY

Spinal Muscular Atrophy (SMA) is a rare neuromuscular disorder characterized by the progressive loss of motor neurons and muscle wasting. Its onset is typically in infancy or early childhood, and if left untreated, it stands as the primary genetic cause of infant mortality. The disease can also manifest later in life with a milder progression. Regardless of the timing, the hallmark is the progressive weakening of voluntary muscles, with an initial impact on the arm, leg and respiratory muscles. Complications may accrue as impaired head control, swallowing difficulties, scoliosis and joint contractures.^56^, ^57^ SMA comes from an anomaly in the survival of the motor neuron protein (SMN1) gene, which encodes SMN, a pivotal protein for motor neuron survival. This genetic flaw results in motor neuron loss within the spinal cord, disrupting communication between the brain and skeletal muscles. An additional gene, SMN2, acts as a disease‐modifying factor, as its copy number influences disease severity. However, due to a nucleotide transition, SMN2 typically produces an insufficient level of functional SMN protein. Human chromosome 5 harbours SMN1 and SMN2 genes at 5q13, with the latter encoding a less effective protein. In SMA, mutated SMN1 fails to produce SMN protein, while most individuals possess at least one functional SMN2 copy, allowing for minimal protein production. Nonetheless, decreased SMN protein availability over time leads to the gradual degeneration of motor neurons. This results in reduced innervation of skeletal muscles, causing progressive atrophy.^58^, ^59^

The clinical manifestation of SMA varies based on the compensatory capacity of SMN2 genes. While the number of SMN2 copies plays a role, other factors influence symptom severity. Management approaches depend on the SMA subtype, with the most severe forms necessitating prompt intervention. Diagnosis relies on genetic testing, often revealing autosomal recessive inheritance or de novo mutations. Approximately 1 in 6000–10,000 babies globally are born with SMA.^60^

Zolgensma® (Onasemnogene abeparvovec) is a gene therapy medication that employs adeno‐associated virus type 9 (AAV9) to deliver the SMN1 transgene to motor neurons. Administered intravenously, Zolgensma® introduces a functional copy of the SMN gene, mitigating disease progression.^61^ Novartis Gene Therapies explore intrathecal administration to expand treatment options. While Zolgensma® is promising, concerns about liver toxicity and adverse events have emerged.^62^, ^63^ Despite these challenges, Zolgensma's widespread adoption marks a significant milestone in SMA management. Zolgensma's development at The Ohio State University's Center for Gene Therapy underscores its transformative potential.^64^ Despite its high cost, Zolgensma® has gained international approval, revolutionizing the approach to treating SMA.

An alternative treatment for SMA is Nusinersen, marketed under the brand name Spinraza®. Nusinersen is not classified as a gene therapy drug like Zolgensma® but is an antisense oligonucleotide (ASO) treatment.^65^ ASO therapies work by modifying the splicing of RNA to increase the production of functional SMN protein in SMA patients. Another ASO therapy used in the treatment of SMA is Risdiplam, which is marketed under the brand name Evrysdi®.^66^ Like Nusinersen, Risdiplam works to increase the production of SMN protein, thereby improving motor function in individuals with SMA.

These ASO therapies represent an essential alternative to gene therapy for SMA and have demonstrated effectiveness in clinical trials, offering different treatment options for patients with this rare genetic disorder. The choice between Zolgensma®, Nusinersen, Risdiplam or other treatments depends on factors such as the patient's age, disease severity and individual medical considerations. As research continues and therapies evolve, the future holds the promise of further improving the lives of those battling SMA and advancing our understanding of genetic diseases.

## 10 ZYNTEGLO®: REVOLUTIONIZING BETA‐THALASSEMIA MANAGEMENT THROUGH GENE THERAPY INNOVATION

Beta‐thalassemia is a genetic blood disorder caused by mutations in the beta‐globin gene. Haemoglobin production is reduced or absent, leading to a variety of symptoms, ranging from anaemia and fatigue to jaundice, organ damage and skeletal abnormalities in severe cases, depending on the specific gene mutations. The deficiency in new beta‐chains leads to the underproduction of adult haemoglobin (HbA), causing microcytic anaemia. Significantly, severe forms of beta‐thalassemia can lead to conditions such as liver cirrhosis, fibrosis and even cancer. It's worth noting that beta‐thalassemia has an exceptionally high prevalence in the geographical region known as the ‘thalassemia belt,’ which spans across the Mediterranean, Middle Eastern and Southeast Asian areas.^67^

Severe beta‐thalassemia, known as transfusion‐dependent thalassemia (TDT), necessitates lifelong regular red blood cell transfusions. Left untreated, TDT damages organs, leading to potential mortality. Diagnosed typically in infancy, TDT stems from impaired beta‐chain haemoglobin production, resulting in chronic anaemia. Patients relying on regular transfusions spend hours in hospitals every few weeks, battling iron overload. Transfusions and chelation therapy alleviate symptoms yet fail to address the underlying genetic cause. Allogeneic haematopoietic stem cell transplantation (allo‐HSCT), the sole potential cure, faces limitations due to donor availability and severe complications.^68^, ^69^

Ex vivo gene therapy utilizing autologous HSCs, like Zynteglo®, emerges as a curative option. Zynteglo® is a gene therapy treatment that utilizes autologous CD34+ cells enriched with lentiglobin BB305 LVs encoding the beta‐A‐T87Q‐globin gene.^70^ This ex vivo therapy offers the potential for functional HbA production, potentially obviating the need for regular transfusions. Administered via a LVs, Zynteglo® delivers functional genes into blood stem cells, avoiding HIV infection.^71^

Phase 3 trials involving 41 patients demonstrated Zynteglo's efficacy in achieving transfusion independence, with up to 89% of patients no longer requiring transfusions. Encouraging results over 24 months were observed, with long‐term follow‐up confirming sustained effects. Despite some side effects, including low platelets and white blood cells, Zynteglo® represents a promising breakthrough in beta‐thalassemia management.^72^ Developed by Bluebird Bio, Zynteglo® earned breakthrough therapy designation from the FDA. Priced at $2.8 million, Zynteglo® marks the third FDA‐approved gene therapy for inherited diseases.^73^ Zynteglo's remarkable progress underscores the significant strides made in gene therapy research and application. Although challenges remain, including side effects and accessibility considerations due to its cost, Zynteglo® symbolizes the promising fusion of science and hope, offering those affected by beta‐thalassemia a renewed chance for a more liberated and improved quality of life.

## 11 HEMGENIX®: A REVOLUTIONARY BREAKTHROUGH IN HAEMOPHILIA B TREATMENT—FDA APPROVAL AND ACCESSIBILITY CHALLENGES

Haemophilia is a genetic disorder that is primarily inherited through families. It severely impairs the body's clotting ability, resulting in an extended duration for bleeding to stop after an injury. Those with haemophilia experience a heightened risk of internal bleeding and excessive bruising. If left untreated, severe cases can lead to permanent injuries in the joints and brain, often resulting in cognitive issues. There are two principal variations of haemophilia: haemophilia A and haemophilia B. Haemophilia A is characterized by low clotting factor VIII levels, while haemophilia B is caused by low clotting factor IX levels. These genetic conditions are typically inherited through X chromosomes, carrying non‐functional genes.^74^ Haemophilia A affects approximately 1 in 5000–10,000 people, while haemophilia B affects around 1 in 40,000 males at birth. Due to its X‐linked recessive inheritance, males are more severely impacted by haemophilia A and B.^75^

Hemgenix® is a gene therapy that enhances factor IX production for haemophilia B patients. It uses AAV5 to carry a modified Factor IX gene variant to liver cells, resulting in more active factor IX.^76^ Hemgenix's efficacy in reducing bleeding incidents is established. It treats severe and moderately severe haemophilia B and has demonstrated substantial reductions in bleeding episodes. On 22 November 2022, the US FDA approved Hemgenix®, marking the first gene therapy for haemophilia B. Hemgenix's single‐dose treatment, priced at $3.5 million, has shown potential to provide long‐term protection against bleeding. Despite its effectiveness, the cost raises concerns about accessibility, particularly in low‐ and middle‐income countries.^77^

In the pursuit of advancing gene therapies for haemophilia, significant challenges emerge. Haemophilia A, being more widespread than B, demands higher factor VIII levels to achieve therapeutic effectiveness. Moreover, the immune responses triggered by viral vectors present hurdles, placing constraints on the potential for repeated administration of gene therapy.^78^ These obstacles underscore the complexity of developing enduring solutions for this bleeding disorder.

## 12 ADSTILADRIN®: GENE THERAPY FOR HIGH‐RISK BCG‐UNRESPONSIVE NMIBC

Bladder cancer, with its origin in the inner lining of the bladder, presents a spectrum of challenges that vary based on its classification and extent of spread. Non‐muscle‐invasive bladder cancer (NMIBC) constitutes the most prevalent form, comprising 70%–80% of initial diagnoses. Often characterized by small mushroom‐shaped growths known as papillary bladder cancer, NMIBC is typically confined to the inner lining of the bladder, making surgical removal of these growths an effective treatment. However, certain NMIBC variants, such as carcinoma in situ (CIS) and high‐grade T1 tumours, pose a higher risk of recurrence due to their rapid growth.^79^

In response to the limitations of current treatments, Adstiladrin® (Nadofaragene firadenovec; rAd‐IFN/Syn3; Instiladrin) has emerged as an innovative gene therapy for high‐risk BCG‐unresponsive NMIBC patients. This groundbreaking therapy aims to introduce a copy of the human interferon‐alfa 2b (IFNα2b) gene to the bladder urothelium. By enhancing the body's anti‐tumour defences through transient local expression of IFNα2b protein, Adstiladrin® offers promise for individuals with high‐risk BCG‐unresponsive NMIBC, whether or not they have papillary tumours. Administered intravesically, Adstiladrin® is supplied in single‐use vials and administered every 3 months via a urinary catheter at a recommended concentration of 3×10^11^ vp/mL.^80^, ^81^

On 16 December 2022, a significant milestone was achieved in the field of bladder cancer treatment as the U.S. FDA granted approval to Adstiladrin®, developed by Ferring Pharmaceuticals. This approval was based on robust data from a multicenter clinical study involving 157 patients with high‐risk BCG‐unresponsive NMIBC, including 98 with evaluable BCG‐unresponsive CIS cases. Adstiladrin®, administered every 3 months for up to 12 months, demonstrated a remarkable 51% complete response rate at 3 months, with approximately half of these responders maintaining their response for over a year. The median response duration was 9.7 months. It's important to note that Adstiladrin® is not recommended for immunosuppressed or immune‐deficient individuals.^82^ These groundbreaking developments in gene therapy represent a transformative step forward in the treatment of high‐risk NMIBC, offering hope to patients who have exhausted traditional treatment options and providing a potential new avenue for the long‐term management of this challenging condition.

## 13 ADVANCING GENOME EDITING: PROGRESS, ETHICAL DILEMMAS AND FUTURE PROSPECTS

**FIGURE 4:**
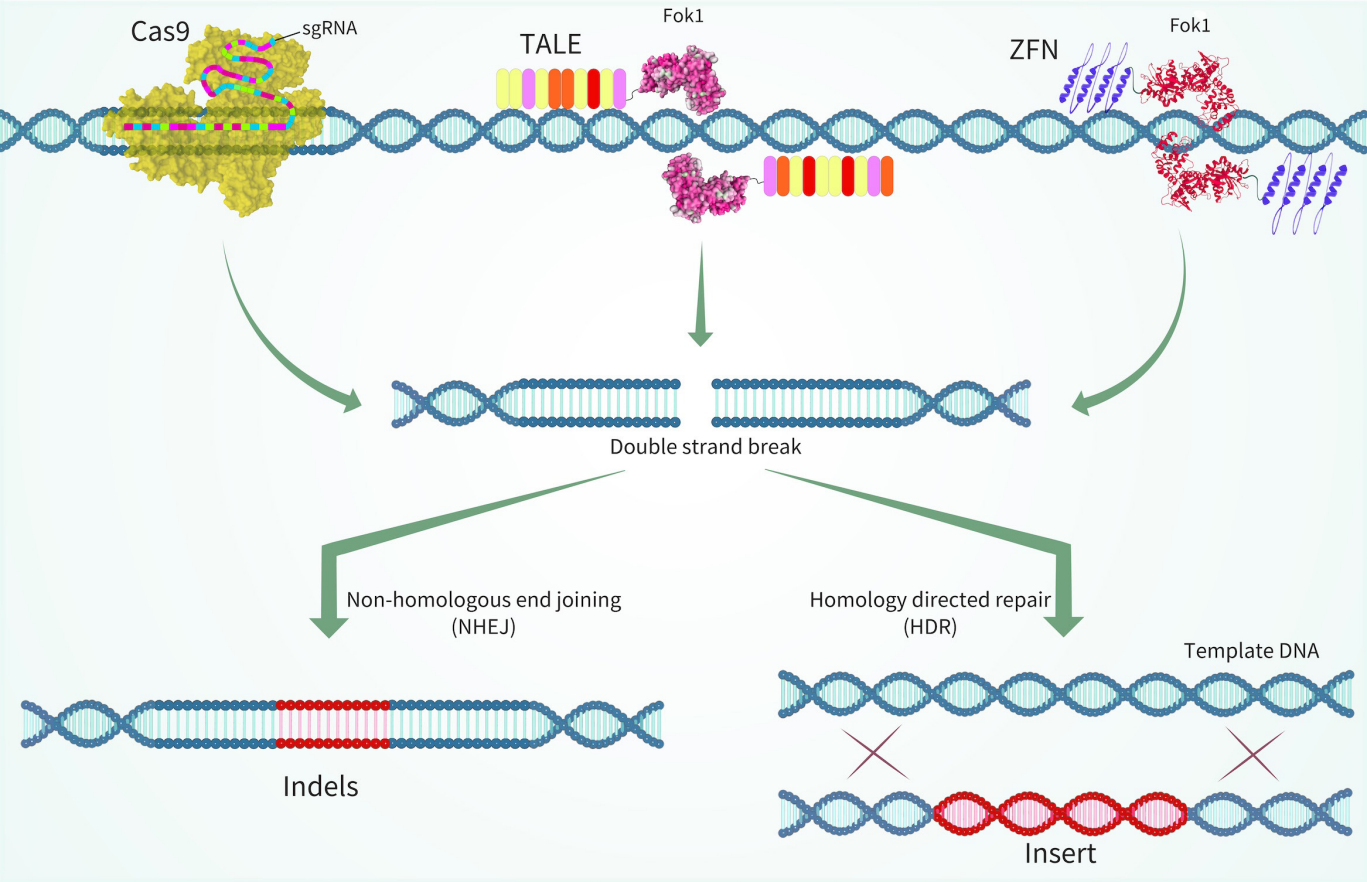
Genome editing molecules, DNA repair mechanisms, and altered genomic outcomes.^83^ Engineered nucleases allow deliberate induction of site‐specific double‐stranded DNA breaks within cellular genomes. Examples include ZFNs, TALENs and Cas9, known as sequence‐specific DNA cleaving tools for genome manipulation. In genome editing processes, natural repair pathways of cells are harnessed for the mending of double‐stranded DNA breaks. The non‐homologous end joining (NHEJ) pathway often yields indel mutations at the repaired DNA break site. The homology‐directed repair (HDR) pathway employs a nucleic acid template for precise repair. In the context of genome‐editing endeavours, investigators can provide a repair template with homologous regions flanking the DNA break, guiding the HDR pathway to generate specific sequence alterations or insertions at the desired genomic locus.

Genome editing, also known as gene editing, has become a vital tool in preventing and treating human diseases. It involves using customizable tools to modify, insert or delete specific parts of our DNA. Among these tools, zinc‐finger nucleases (ZFNs), transcription activator‐like effector nucleases (TALENs), and the RNA‐guided CRISPR‐Cas nuclease system (RGENs) are widely recognized (Figure 4). These technologies create breaks in the DNA at targeted locations, which are then repaired through processes like homologous recombination (HR) or non‐homologous end‐joining (NHEJ), often with the assistance of donor DNA.^84^ When comparing these genome editing techniques, notable distinctions emerge. ZFNs and TALENs, as man‐made approaches, necessitate the intricate design of custom proteins to achieve precise DNA cleavage (Table 3). In contrast, CRISPR‐Cas, derived from a natural bacterial defence system, utilizes small RNA molecules to guide Cas proteins, offering a more adaptable and straightforward method. Clustered Regularly Interspaced Short Palindromic Repeats' (CRISPR's) versatility is particularly evident, as it can simultaneously target multiple genes, whereas ZFNs and TALENs may require more effort for multi‐gene editing tasks. The simplicity and accessibility of CRISPR have made it a dominant player in genome editing applications, raising ethical and safety concerns.^80^, ^82^

**TABLE 3:** Comparative overview of programmable nucleases for genome editing [83, 85].

	Zing‐finger nucleases (ZFNs)	Transcription activator‐like effector nucleases (TALENs)	Clustered regularly interspaced short palindromic repeats‐CRISPR‐associated‐9 (CRISPR‐Cas9)
Target sequence recognition	Zing‐finger proteins	Transcription activator‐like effectors	crRNA or sgRNA
Endonuclease	*FokI*	*FokI*	Cas9
DNA sequence recognition size	18–36 bp	30–40 bp	17–20 bp + NGG
Specifity (off‐target)	High	Low	Variable
Constraints in design & targeting	Assembly of zinc‐fingers is context‐dependent, with a preference for GC‐rich target sequences	Assembly of TALE repeats yields a 5′‐targeted thymine base	PAM requirement (NGG for SpCas9)
Repair mechanism	Non‐homologous end joining	Homology‐directed repair	Non‐homologous end joining
Targeting efficiency	Low	Moderate	High
Advantage	Small protein size enables easier ex vivo and in vivo introduction into cells, with low immunogenicity due to the use of human protein scaffolds in zinc fingers	The lack of context dependence, leading to a more straightforward assembly process	Offers high multiplexing efficiency, ease of retargeting through cloning and oligo synthesis, reliant on predictable Watson‐Crick base‐pairing
Drawbacks	The necessity for protein engineering for re‐targeting, high production costs, absence of direct correspondence between amino acid sequence and DNA recognition sites, context‐dependent specificity leading to off‐target effects	Complex molecular cloning (utilizing Golden Gate Assembly) for production, and their large protein size which could pose difficulties in ex vivo and in vivo cell introduction	A higher frequency of off‐target events compared to ZFNs and TALENs, potential risk of immunogenicity, and limitations in target selection due to the requirement for specific PAM sequences

*Note*: This table offers a comprehensive comparison of programmable nucleases used in genome editing. Programmable nucleases are molecular tools capable of introducing precise modifications into DNA sequences. The table presents a side‐by‐side analysis of different nuclease platforms, including CRISPR‐Cas9, ZFNs (Zinc Finger Nucleases), and TALENs (Transcription Activator‐Like Effector Nucleases). Parameters such as specificity, efficiency, repair mechanism, and advantages/disadvantages are compared, providing valuable insights into the strengths and limitations of each nuclease technology.

CRISPR is a groundbreaking genetic technology that allows precise modification of DNA by targeting and editing specific genes or sequences within the genome. These systems store foreign DNA sequences as spacers, resulting in unique CRISPR RNAs with distinct spacers. The RNAs guide Cas proteins, such as Cas9 in Type II CRISPR‐Cas, to specifically cleave invasive DNA, using a protospacer adjacent motif (PAM) as a recognition signal. This unique feature has transformed CRISPR into a versatile genome editing platform.^86^ Renowned for its speed, affordability, accuracy and efficiency, CRISPR/Cas9 is widely employed in genome engineering, scientific research and drug trial evaluations. Its multifaceted significance in advancing genetics and potential therapeutic applications is increasingly evident.^87^

Recent innovations in CRISPR have opened new possibilities in precision medicine, with ongoing trials covering seven areas: blood disorders (particularly sickle cell disease (SCD) and β‐thalassemia), cancers, inherited eye diseases, diabetes, infectious diseases, inflammatory conditions and protein‐folding disorders. As this technology advances, its potential to improve medical treatments and human health is becoming increasingly clear.^85^ However, using genome editing, especially CRISPR/Cas9, in altering the human genetic code has raised ethical concerns. Most genome edits are limited to non‐heritable, tissue‐specific changes in somatic cells, excluding egg and sperm cells. The ethical debate mainly revolves around the permissibility of using these technologies to enhance normal human traits, particularly when applied to germline cells or embryos. Due to these ethical and safety concerns, many countries, including the United States, currently prohibit germline and embryo genome editing.^87^, ^88^

FDA is overseeing a growing number of CRISPR clinical trials, although most are still in their early stages. Particularly, landmark trials on SCD and β‐thalassemia that were conducted in Germany and the United States, have demonstrated positive outcomes. Patients have experienced remarkable recoveries, marked by near‐normal haemoglobin levels and reduced depenence on transfusions.^89^ On 8 December 2023, FDA approved Casgevy, the first therapy using CRISPR/Cas9 for SCD, developed by Vertex Pharmaceuticals and CRISPR Therapeutics. On the same day, FDA announced the approval of another gene therapy drug named Lyfgenia. Developed by Bluebird Bio Inc., Lyfgenia uses LVs to introduce functional beta‐globin gene copies, offering a personalized treatment approach for SCD patients.^90^ While both products are promising, CRISPR‐based treatments for blood disorders face scalability and cost challenges. The expense of current therapies limits accessibility, and the technology's broad application requires refinement and optimization.

Collectively, these developments highlight the transformative potential of CRISPR technology in revolutionizing the treatment landscape. The ongoing trials illuminate avenues for therapeutic innovation, holding the promise of improving the lives of millions worldwide. However, the novelty of CRISPR treatments highlights the need for ongoing vigilance, especially in assessing treatment durability and unintended edits. Recent advancements include multiplex editing, base editing, and prime editing, offering versatile options for precise genetic modifications. This impressive progress in isolated cells and animal models, particularly in cancer immunotherapy, underscores the potential of multiplex editing.^89^, ^91^ As CRISPR‐based interventions evolve, the landscape is poised for transformative developments, reshaping the future of medicine and offering hope for novel therapies.

## 14 CONCLUSION

In summary, gene and cell therapy represent revolutionary fields in medical science, offering unprecedented opportunities to address previously incurable diseases at their root. These cutting‐edge therapies hold the promise of personalized and precise treatments, harnessing the power of genetic and cellular manipulation to correct or replace faulty genes and cells. As research and technological advancements continue to accelerate, the potential impact of gene and cell therapy on healthcare is immense. While challenges and ethical considerations persist, the remarkable progress made thus far suggests a transformative future where these therapies become integral components of mainstream medicine, providing hope for patients and paving the way for a new era in healthcare.

## AUTHOR CONTRIBUTIONS

**Busra Cetin:** Investigation (equal); writing – original draft (equal). **Fulya Erendor:** Investigation (equal); writing – original draft (equal). **Yunus E. Eksi:** Investigation (equal); writing – original draft (equal). **Ahter D. Sanlioglu:** Investigation (equal); writing – original draft (equal). **Salih Sanlioglu:** Investigation (equal); supervision (equal); writing – original draft (equal).

## FUNDING INFORMATION

This research received no specific grant from any funding agency, commercial or not‐for‐profit sectors.

## CONFLICT OF INTEREST STATEMENT

The authors confirm that there are no conflicts of interest.

## CONSENT FOR PUBLICATION

All authors have read and approved the final version of the manuscript and its publication in the Journal of Cellular and Molecular Medicine.

## Data Availability

Data derived from public domain resources.
